# Hemeralopia

**Published:** 1854-11

**Authors:** C. S. Fenner

**Affiliations:** Memphis, Tenn.


Hemeralopia. By C. S. Fenner, M. D., of Memphis, Tenn.—June, 1847, a servant boy, a field hand, 24 years of age, was brought me with hemeralopia. During the day his sight was perfect with no abnormal appearance of the eye; the irides acting freely to the stimulus of light. On examining his eyes by candle light, the pupils were found to be as widely dilated as they usually are after the continued application of stramonium or belladonna. The lighted candle was brought within two or three inches of the eyes without producing the least contraction of the pupils or causing the slightest perception of light. He remained in this condition until sunrise the next morning, when his sight began to return, and in a few minutes became perfect, continuing so through the day. The same symptoms had recurred nightly for a month previous to my seeing him, without interfering with his field labors during the day. As there seemed to be a slight determination of blood to the head, with an unusual fulness of the pulse, I took a few ounces of blood from the arm, prescribed a mercurial carthartic, and applied a large blister to the back of the neck; and the next day gave ten grains of quinine. The second night the blindness returned at the usual time and continued some four or five hours; after midnight the patient was able to see distinctly by the light of the moon. Continued a succession of blisters to the temples and mastoid. The fourth night the loss of sight lasted about an hour, after which time there was no return of the disease, and at the end of the week the patient was discharged cured.

Since the above mentioned patient was treated, I have seen and been consulted in a considerable number of similar cases, all them occurring among negroes working in an extended range of prairie plantations, commencing in Pickens county, Alabama, and extending up through Noxubee, Lowndes, Oktibbeha and Monroe counties, Mississippi.

The disease makes its appearance almost invariably in the spring, or early in the summer months, before the young corn and cotton plants have attained sufficient size to shield the eye from the dazzling reflection of the sun's rays from innumerable small pieces of limestone, and fragments of shells that are thickly interspersed through the soil. These prairies constitute immense bodies of a peculiar black soil, lying on a strata of limestone. In many places the rock approaches the surface, and is entirely bare, reflecting the sun so powerfully as to be almost insupportable to the eye. To a person standing in the middle of some of these large prairies, the horizon, in many directions, seems to meet the surface without a tree or shrub to obstruct the view. I attribute the frequent occurrence of night-blindness through this section of country, to the exhaustion of the nervous power of the retina from continued exposure day after day to the powerfully reflected solar rays, without having any objects, even at a distance, (particularly trees covered with green foliage,) to relieve the eye, by frequently looking up and bringing a change of scenery within the field of vision.

All the cases of hemeralopia that I have seen, have readily yielded to treatment similar to that pursued with the patient above mentioned. In a majority of instances bleeding is unnecessary. Counter irritation from a succession of blisters, and repose within doors for a few days, are usually all that is requisite to give entire relief. I have been informed of several cases that recovered after the lapse of a few months, without any treatment whatever; and, in no instance have I known any permanent injury to vision result from permitting the disease to take its own course. Cauterizing the margin of the cornea, with nitrate of silver, has been recommended in hemeralopia, but as this method of treatment is much more painful than blisters, and as the relief from the latter is speedy and effective, I give them the preference. —*New Orleans Med. and Surg. Journal.*

